# Effect of Motorized Elephant-Assisted Therapy Program on Balance Control of Children with Autism Spectrum Disorder

**DOI:** 10.1155/2019/5914807

**Published:** 2019-11-18

**Authors:** Satiansukpong Nuntanee, Sasat Daranee

**Affiliations:** ^1^Department of Occupational Therapy, Faculty of Associated Medical Sciences, Chiang Mai University, Thailand; ^2^Elephant Assisted Therapy Project, Department of Occupational Therapy, Faculty of Associated Medical Sciences, Chiang Mai University, Thailand

## Abstract

Children with autism spectrum disorder (ASD) have poor balance, and this limitation has effects on their daily living activities. The purpose of this study was to create the motorized elephant-assisted therapy program (METP) and examine the effect of the METP on balance control improvement in individuals with ASD. Twenty participants, aged 8 to 19 years, were recruited from occupational therapy clinics around Chiang Mai city and were divided into 2 groups: control and experimental. Participants' balance control was tested by measuring their postural sways in a bipedal stance by using a Swaymeter under four conditions: “floor-eyes open,” “floor-eyes closed,” “foam-eyes open,” and “foam-eyes closed.” Pretests were administered one week before participation in the METP, and then, posttests were administered one week after completing the METP. Each participant took a 1.5-hour session of the METP, twice a week for a 4-week duration. In one session, 2 participants were assigned to work with two motorized elephants in 4 activities: washing the elephant, climbing up and down the elephant, riding the elephant, and playing a game while riding the elephant. Results showed that the pretest control and experimental groups were not significantly different in their balance control, but at posttest, the postural sway of the experimental group was significantly different from that of the control group in two conditions: floor-eyes open and floor-eyes closed. Their lesser anteroposterior range of postural sway showed that the experimental group gained balance control improvement. In conclusion, the finding of this study showed that the METP could be an alternative treatment method to facilitate better balance control in individuals with ASD.

## 1. Introduction

The effect of engaging individuals with ASD in the METP is a part of a larger study exploring the effect of this program on social communication, social interaction, and balance of children with autism spectrum disorder (ASD). It is the balance aspect that is addressed in this paper.

A high prevalence of motor impairment has been found in children with ASD [1]. Despite motor impairments in autism spectrum disorder (ASD) being widely reported and acknowledged [2], impairments in social communication and social interaction are drawing more attention in treatment [3–5]. Recently, many more studies have found a correlation between balance performance of ASD and the ASD symptom severity [5, 6].

The presence of diverse motor impairments in individuals with ASD includes poor motor anticipation, motor pattern disturbances, clumsiness, impaired postural control and postural stability, dyspraxia, and impaired gross and fine motor movement [3, 4]. Standing balance is noted as a consistent area of concern in individuals with ASD [5–8]. Balance of many individuals with ASD has never reached an adult level like typical individuals [9]. Poor balance control or postural control expressed by postural sway in a stance with both legs and with single leg is found in children with ASD when compared with typically developing children [6].

Adequate balance is necessary for performing daily living activity such as dressing, bathing, and engaging in leisure activities such as sport and riding a bicycle [10]. Poor balance can prevent children with ASD to participate in social and leisure activities such as dancing and playing a sport such as badminton and cycling. Balance is more challenging in children with ASD. A previous study suggested hypotonia as one factor that may cause poor balance control in children with ASD. This was exhibited in the single leg balance task which may be linked to the impairment of the basal ganglia function [10, 11]. Fifty-one percent of 154 children and adolescents with ASD exhibited hypotonia [12].

Good control of body movements emerges from the interaction of three important systems: the person, activity or task, and environment. Other factors within the person such as sensory, motor, perceptual, cognitive, emotional, and other physical aspects related to the neurological, musculoskeletal, and cardiovascular systems of the body are related as well [13]. Good balance control both in static and dynamic positions requires good support from multiple sensory systems: visual, proprioceptive, and vestibular which provide feedback for motor corrections [14].

Balance is the ability to keep the centre of mass in the base of support. It helps us to keep the body in the desired position for as long as we need. The balance control mechanism is the ability of neuromuscular activity to maintain and keep the projected centre of mass in the base of support [15]. Balance performance can be increased by using training programs that involve sensory system manipulation, e.g., limited visual information and varying floor stability [16]. It can also be improved by increased task demands, environmental constraints [17], and physical experience [18].

Previous studies using the elephant-assisted therapy program (TETP) showed balance performance improvement in those participants with ASD and Down syndrome [19, 20]. However, there were some limitations using the TETP such as high cost of treatment, long travel distance, and accessibility. Prior studies showed that the owner of artificial pets or animal robots could interact and create bonding with these robots [21]. It assisted in reducing anxiety and in promoting self-calming. It also reduced the overwhelming complexity of person to person interactions [21, 22]. Varughese confirmed that the animal robot could reduce both the physical and emotional stress in the same way as when using a real animal [22]. In addition, animal robots could be used with clients who have limited access to real animals and who have allergic symptoms when being with a real animal or if the person has physical weakness or anxiety due to feeling unsafe [23].

The motorized elephant-assisted therapy program (METP) was created and designed by three occupational therapists in order to provide meaningful activities and physical experience and a play environment for individuals with ASD. The purpose of this study was to examine the effect of the METP on balance control improvement in individuals with ASD by using a Swaymeter. The following research questions guided the current study: (1) Do the balance controls of the control and experimental groups differ at pretest? (2) Do the balance controls of the control and experimental groups differ at posttest?

## 2. Materials and Methods

### 2.1. Research Design

This is a quasi-experiment, with a two groups pretest and posttest design. Participants were voluntarily assigned into a control group and an experimental group. Both groups received regular standard occupational therapy treatment. The experimental group received additional 8 treatment sessions of the METP. Pretests and posttests were administered one week before and readministered after completion of the METP. All procedures for the study were approved by the Human Research Ethics Committee, Faculty of Associated Medical Sciences, Chiang Mai University. Parental consent and participants' assent forms were obtained prior to the start of the intervention.

### 2.2. Participants

Twenty participants diagnosed with ASD, aged 8 to 19 years, were purposively recruited from occupational therapy clinics around Chiang Mai city. Participants were divided into control and experimental groups. Age and gender were matched in pairs. There were 9 males and 1 female in each group. Inclusion criteria included those with ASD, having poor balance scores based on the results on the balance subtest of the Bruininks Oseretsky Test of Motor Proficiency (BOT-2). Inclusion criteria also required the participants to be capable of communicating their own needs and having self-care independence. Exclusion criteria included those who were unable to commit to participate in 8 METP treatment sessions. Age means and standard deviation between control and experimental groups were 11.81 ± 2.95 and 12.15 ± 2.47 years, respectively. The average age between groups was not significantly different (*t* = 0.280, *p* = 0.78).

### 2.3. Instrument

**Figure 1:**
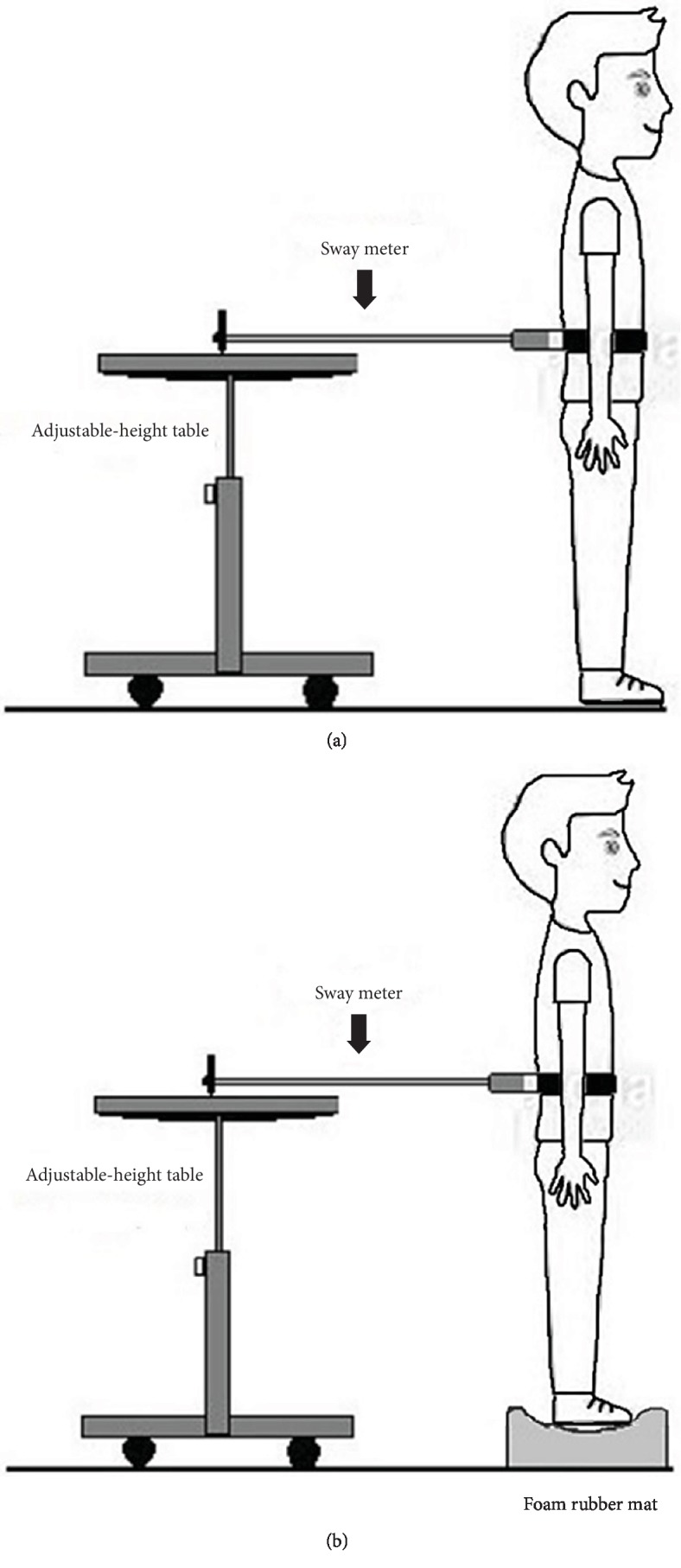
Postural sway tests: (a) standing on the floor and (b) standing on a foam rubber mat.

A Swaymeter is a useful clinical test because it can be used in a variety of settings and requires a short time to administer. A Swaymeter used in this study (see Figure 1) is a subtest of Professor Stephen Lord's Physiological Profile Assessment (PPA) of the Neuroscience Research Australia formerly known as POWMRI FallScreen®, the Prince of Wales Medical Research Institute [24]. The test-retest reliability of sway on floor eyes open, sway on floor eyes closed, sway on foam eyes open, and sway on foam eyes closed were 0.68, 0.85, 0.57, and 0.83, respectively [24].

The Swaymeter is feasible for use in a diverse population: children, adult, and elderly [25, 26]. Sway displacement measures are used to indicate good and poor balance controls. Good agreement between the Swaymeter and force plate for measuring anteroposterior (AP) and mediolateral (ML) ranges was found. Moderate to good correlations with force plate results across conditions (*r* = 0.560 − 0.865) have been reported [27].

The balance subtest of the Bruininks Oseretsky Test (BOT2) [28] is an eight-item scale that measures specific balance skills. The test-retest reliability within 7-12 days for the balance was 0.56.

**Figure 2:**
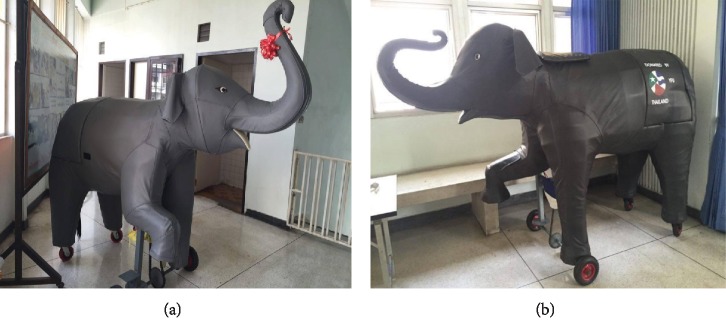
(a, b) Two motorized elephants used in the METP.

Two motorized elephants (see Figure 2) were made by a worker under the supervision of an elephant expert, Prasop Tipprasert.

The motorized elephant-assisted therapy program (METP) was created by three occupational therapists who were experienced in using TETP for children with ASD for more than 5 years. The METP used 2 motorized elephants, and the treatment media was composed of 4 activities: washing the whole body of the motorized elephant, getting on and off, riding, and playing games.

**Figure 3:**
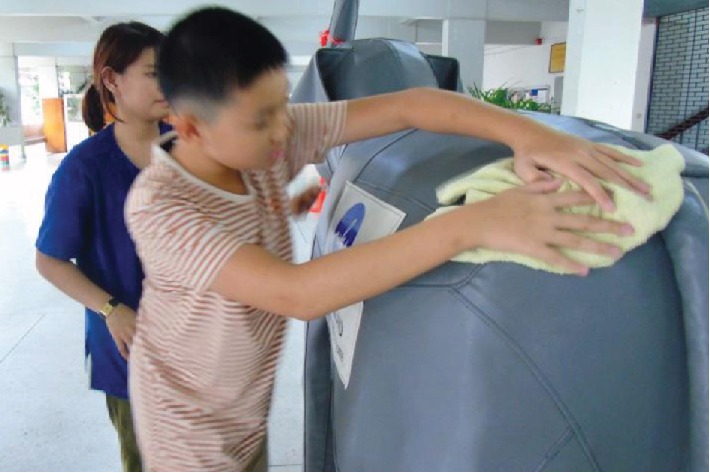
Washing the elephant's back.

In the washing activity (see Figure 3), participants first learned to soak a small towel in water and then squeeze the water out. Second, they used a wet towel to clean the skin surface of the motorized elephant. While washing, participants bent their body and were also required to step up and down a 2-step stair to reach the belly or the head of the motorized elephant. Then, using a dry towel they dried the elephant skin.

To get on the motorized elephant, participants stood on the floor on the right side of the elephant, used their right hand to grab the right ear, and then put their right foot on the right leg of the elephant. Their left leg moved across the elephant's body, and then, they sat on the elephant's neck. To get down from the motorized elephant, participants used their right hand to grab the elephant's right ear and bend their body forward while moving their left leg across and over the elephant's body to the right side. Their body moved down to their right side until their right leg stood on the right leg of the elephant, then using left and right legs alternatively stepped down to the ground.

While riding, participants rode the motorized elephant in line both forwards and backwards. After becoming familiar with the movements, the riding was made more challenging by increasing the complexity of the path. The “elephant's” path was adjusted to backward and forward, circular, rectangular, and diagonal movements.

**Figure 4:**
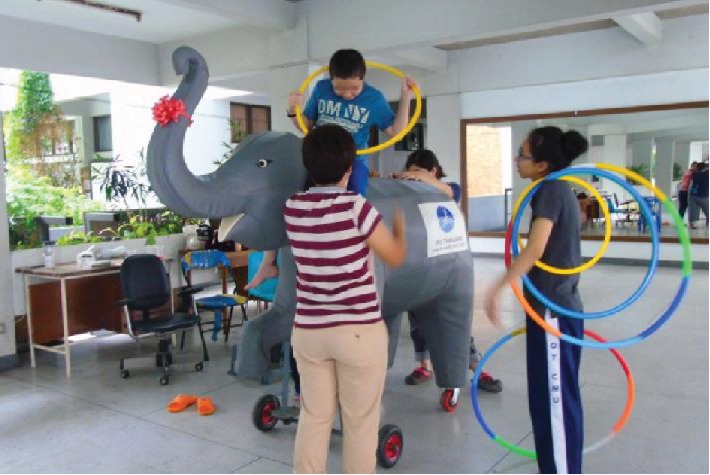
Playing with a hula hoop while riding the elephant.

**Figure 5:**
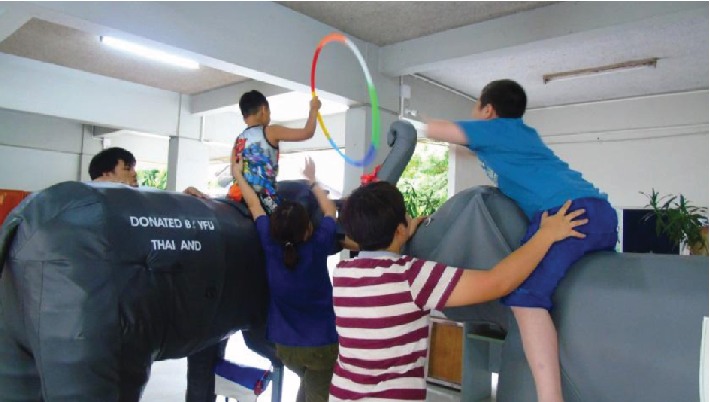
Playing games with a friend.

**Figure 6:**
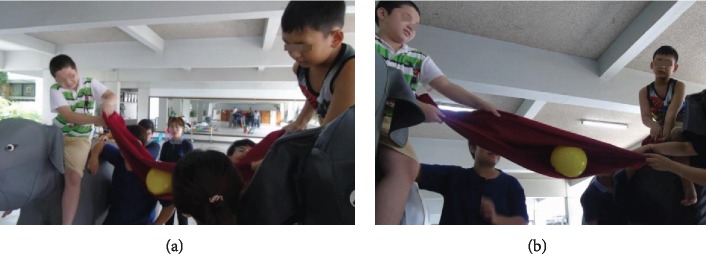
(a, b) Help each other to put the ball into a basket.

Participants played games while sitting on the moving motorized elephant (see Figures 4–6). In some games, players are required to get on and off the elephant from time to time in order to push or pull the elephant as needed. These games were designed to increase balance control as well as to have fun and pleasure while playing with the other participants. Games included throwing a ball into a basket, collecting tickets hung on a ceiling, and putting a chain into a bottle. Each of the two motorized elephants was controlled by each participant to direct its movement: forward, backward, stop, and left and right turns.

### 2.4. Procedures

Balance control was identified by using a Swaymeter to measure the postural sway of each participant during the week before and after the intervention. Postural sway was measured in bipedal stance on bare feet, with eyes open and closed, on a firm surface and on a foam. Data was obtained from the four conditions: floor with eyes open (EO floor); floor with eyes closed (EC floor); foam with eyes open (EO foam); and foam with eyes closed (EC foam). A trial of 30 seconds was conducted for each condition, for a total of 4 trials per participant. Participants were instructed to stand still without talking, with feet shoulder-width apart, while looking forward and slightly down at a 1.5-metre wall.

The Swaymeter measures postural sway at waist level. The device is composed of a 40 cm long rod with a vertically mounted pen at its end. The rod is attached to the participant by a firm belt and extends posteriorly (see Figure 1). As the participant tries to stand as still as possible for 30 seconds, the pen records the postural sway on a sheet of millimetre graph paper fastened to the top of a height-adjustable table. One trial of each condition was performed in the following order: standing on the floor with eyes open, on the floor with eyes closed, on the foam with eyes open, and on the foam with eyes closed. The foam is a medium-density foam rubber mat, 15 cm thick. Anteroposterior and mediolateral sway ranges (AP and ML) were recorded for the 4 conditions. The postural sway area in each condition was calculated by multiple of AP with ML [24].

For treatment, a session of the METP was run by a team of occupational therapists and occupational therapy students. Each experimental group participant worked with one occupational therapy professional at each treatment session twice a week, continuously for a 4-week duration. The length of each session was 1 hour and 30 minutes: 10 minutes for introduction, 20 minutes for washing and towel drying the elephant, 20 minutes for riding and controlling the elephant to go to the right direction following a command, 30 minutes for playing a game with peers, and 10 minutes for wrapping up and putting away the materials.

### 2.5. Data Collection and Analysis

The postural sway was recorded in anteroposterior (AP) and mediolateral (ML) sway range. Postural sway area (mm^2^) was calculated from multiple AP and ML. For descriptive statistics, means and standard deviations were calculated. For inferential statistics: multivariate analysis of variance (MANOVA) was utilized to compare postural sways between groups at pretest as well as at posttest. Further within-group analysis between pretest and posttest was done using paired*t*-test.

## 3. Results

This study used a Swaymeter to examine the improvement of balance control in children with ASD after receiving the motorized TETP. The shorter the postural sway range and the smaller the sway area, the better the balance control performance. Mean and standard deviation of postural sway (anteroposterior (AP) range and mediolateral (ML) range) and sway area under four conditions in control and experimental groups at pretest and posttest are shown in Table 1. At pretest, the control and experimental groups showed no significant difference of postural sways (*p* > 0.05) in AP range, ML range, and the sway area in all four conditions as shown in Table 2. At posttest, the control and experimental groups showed significant differences in postural sways under two conditions: floor-eyes open and floor-eyes closed in the AP range, but no differences in the other two conditions: foam-eyes open and foam-eyes closed. A further analysis to see balance control improvement in the control group by using paired *t*-test revealed no significant improvements between pretest and posttest in postural sway ranges (AP, ML) and the sway areas in four conditions with eye opened and eye closed (see Table 3). However, further analysis to see balance control improvement in the experimental group (see Table 4) found significant improvement between pretest and posttest: (1) AP sway range on floor-eyes open, (2) ML sway range on floor-eyes closed, (3) ML sway range on foam-eyes open, and (4) AP sway range on foam-eyes closed. The posttest sway areas were significantly smaller than the pretest sway areas on 2 conditions: floor-eyes open and foam-eyes open.

Parents observed their children doing real-life activities such as ADL and play. From qualitative results, 60 percent of parents reported that the experimental group gained better postural control and balance in the real-life activities after the treatment.

## 4. Discussion

The aim of this study was to compare patterns of postural sways in individuals with ASD before and after the METP. At pretest, the control group (*n* = 10) and the experimental group (*n* = 10) showed no significant difference in postural sway (*p* > .05) in all four conditions. Both groups exhibited a higher challenge with eyes closed than with eyes open and with an unstable floor than with a stable floor. Therefore, balance control was rated from low to high with eyes open on floor, eyes closed on floor, eyes open on foam, and eyes closed on foam. The AP and ML ranges and sway areas corresponded to the challenges to balance of the task. At pretest, both groups showed poor balance control as shown via their AP and ML ranges and sway areas [9]. Minshew et al. reported that 99 individuals with autism, aged 5-52, had significant difference of postural control from typical individuals [9]. Previous studies reported that individuals with ASD have longer AP and ML and sway areas than typical children [6, 29]. For example, stance with eyes open on floor children with ASD (*n* = 21, aged 9-14 years) showed a mean for the AP range of 34.7 ± 16.8 mm and a mean for the ML range of 48.2 ± 45.5 mm, while typical children (*n* = 30, aged 8-15 years) showed a mean for the AP range of 17.4 ± 6.9 mm and a mean for the ML range of 12.7 ± 6.8 mm [29]. Previous studies showed that children with ASD exhibited a higher amount of sway in most of the parameters such as range, mean velocity, and sway area compared to typical children [26, 29]. Evidence supported that poor balance control in children with ASD was partially due to poor processing and integration of vestibular, somatosensory, and visual sensory inputs [9, 26].

At posttest, the results showed a significant difference in postural sways between the groups in two conditions: EO floor and EC floor, but not in the other two conditions on the foam. In the EO floor and the EC floor tasks, the experimental group had significantly different postural sway from the control group in the AP range. This indicated that their balance control in the anteroposterior direction was better than that of the control group. This may be due to the effect of the METP, which was additionally provided twice a week continuously for 5 weeks for the experimental group.

The METP provides more task demands for participants in the experimental group such as physical activities (washing a towel, going up and down a motorized elephant) and also provides opportunities to get complicated physical experience such as playing a game while riding on the neck of an elephant. All activities of the METP provide meaning, motivation, and sensory information, including tactile, proprioceptive, and vestibular which are factors within an occupational therapy frame of reference [23, 30, 31]. For example, washing a towel before and after washing the motorized elephant provides tactile and proprioceptive information and is considered a purposeful occupation.

The climbing up and down the stairs to mount the elephant and ride the elephant while it was walking in various directions as well as varying the speed provided vestibular input. With the METP, the experimental group may have benefited from this increased sensory experience. Their postural control and balance were facilitated through righting, equilibrium, and protective reactions. These might improve the balance control mechanism of participants in the experimental group as the results showed a trend of decreasing postural sways both for the AP and ML displacement and the sway area.

The METP required participants to plan the sequence of their movement, estimate space and distance, and perform a variety of movements. The motorized elephant can move on the anterior and posterior directions; therapists help to turn the ME on to the left–right directions. The quantitative data confirmed that the participants improved their balance control.

New habits and new roles were developed in the children of the experimental group to perform the relevant elephant-facilitated occupations. Balance controls improved by providing more task demands and environmental opportunities [17, 18]. Hubbard et al. suggested that task-specific training should be relevant, randomly ordered, repetitive, and reconstructed and should involve massed practice of the whole task and be positively reinforced to the patient [32]. For example, washing the elephant required the individual with ASD to climb up small steps in a forward direction and climb down small steps in a backward direction. In playing a game while riding the elephant, participants were required to use their hand to throw a ball, control a chain to put in a bottle, or collect tickets. These task demands and more physical experience could assist participants in gaining balance in the anteroposterior direction.

In the other two conditions, eyes open on foam and eyes closed on foam, the control group showed no improvement in their postural sways in AP and ML ranges and sway areas, but the experimental groups showed an improvement trend. Their AP and ML ranges after the METP were declined, and also, their sway area was significantly smaller than that at pretest. The balance control on an unstable floor (foam) was more difficult than that on a stable floor. The experimental group benefited from the METP. Longer AP and ML ranges and sway areas were found on the unstable floor than the stable floor and with eyes closed than with eyes opened. Further investigation in individuals with ASD who received a longer period of the METP might be interesting.

## 5. Conclusions

The control group showed no balance control improvement. The experimental group had significantly improved in balance control after the METP in the AP range both EO floor and EC floor, but not in the other two conditions: EO foam and EC foam. The finding of this study showed that the METP could be an alternative treatment method to facilitate better balance control in individuals with ASD.

### 5.1. Limitations

This study is a part of a larger study which examines the effect of METP on balance and social performance. The METP was designed to promote both variables. Even though the treatment was based on a one on one basis, the METP was provided with 2 participants at a time. The number of participants in each group, control and experimental, was 10; therefore, the study results have limited generalization to other groups of children with ASD. Further investigation is required. Potentially limited applicability of this intervention might be culture and cost. Thai culture may ease Thai occupational therapist to implement the METP because Thai children are familiar with elephant from song, toys, etc. The extra cost of the motorized elephant might impede intervention implementation.

### 5.2. Implications for Occupational Therapy Practice

Clinical implication from the present results reinforces that occupational therapist should use simple occupations in daily life such as cleaning, washing, and play activities. Occupational-based treatment results are quite good in facilitating functional improvement in children with ASD such as in helping them to be more aware of their environment, to engage with an activity more effectively, and to have the opportunity to improve their body balance control and movement through various physical experiences. Participants with ASD had to change centre of mass and shift weight quite often while engaging the activities in the METP.

Two challenging characteristics of children with ASD are having poor engagement while doing activities and lacking play skill. The METP provides a motorized elephant (ME), a new therapeutic medium, using meaningful tasks and play activities with a peer. The combination of the ME, a meaningful task, and a peer may provide more engaging opportunities for children with ASD. They assist in promoting a different context and a real play situation. These draw more attention and motivation from the children with ASD. Occupational therapists should address play with peers and meaningful tasks in the ASD treatment sessions because play is fun and a meaningful task can be transferred and used in a real-life situation with a meaningful task and play opportunity; it is quite easy for an occupational therapist to change the role of a child and provide more variety of physical exertion and experience. These could assist to promote balance control and alleviate other ASD symptoms

## Figures and Tables

**Table 1:** Postural sway means and standard deviation of control and experimental groups at pretest and posttest.

	Pretest			Posttest		
	Mean ± SD	Min.	Max.	Mean ± SD	Min.	Max.
*Control group* (*n* = 10)						
(1) EO floor						
AP	42.00 ± 21.15	21	95	52.90 ± 25.46	24	111
ML	34.90 ± 21.34	11	82	36.60 ± 15.45	16	63
Area	1497.30 ± 1395.88	363	5225	2044.50 ± 1470.14	576	4662
(2) EC floor						
AP	54.20 ± 22.94	21	86	66.00 ± 25.24	35	119
ML	45.80 ± 35.37	18	137	27.00 ± 10.02	11	43
Area	2761.30 ± 2709.73	396	9590	1733.70 ± 812.61	627	3332
(3) EO foam						
AP	60.80 ± 26.81	28	112	78.20 ± 42.88	45	190
ML	46.20 ± 13.62	23	66	40.60 ± 11.30	25	63
Area	3074.00 ± 2117.87	780	7392	3411.20 ± 3066.23	1150	11970
(4) EC foam						
AP	77.20 ± 30.29	43	134	73.30 ± 26.74	41	118
ML	61.60 ± 16.09	46	99	76.50 ± 33.79	39	136
Area	4928.40 ± 3151.87	2322	3266	5433.60 ± 2692.10	2223	9234
*Experimental group* (*n* = 10)						
(1) EO floor						
AP	48.40 ± 31.45	15	108	26.00 ± 18.24	6	62
ML	43.30 ± 20.50	16	78	35.80 ± 24.32	14	82
Area	2340.90 ± 2039.33	240	6630	1176.80 ± 1388.68	84	3968
(2) EC floor						
AP	45.60 ± 20.93	18	76	38.70 ± 30.56	3	105
ML	50.00 ± 26.67	22	106	39.10 ± 29.56	14	115
Area	2378.50 ± 1724.63	396	6075	1804.40 ± 2162.29	90	5980
(3) EO foam						
AP	62.50 ± 32.86	25	139	48.50 ± 30.15	7	102
ML	55.20 ± 25.33	24	113	34.30 ± 14.26	16	62
Area	3661.40 ± 2562.35	600	8479	1845.00 ± 1463.50	168	3850
(4) EC foam						
AP	72.30 ± 24.40	37	115	56.20 ± 30.81	32	131
ML	75.00 ± 39.95	38	159	65.50 ± 28.72	27	112
Area	5846.00 ± 4874.10	1406	18285	3974.30 ± 3167.48	1053	10349

AP = anteroposterior range in millimetres; ML = mediolateral range in millimetres; Area = sway area in square millimetres.

**Table 2:** Comparison of postural sway between control and experimental groups at pretest and at posttest by using multivariate analyses of variance (MANOVA) at *α* = 0.05.

Postural sway	Control vs. experimental		Control vs. experimental	
	Pretest		Posttest	
	*F*	*p*	*F*	*p*
(1) EO floor				
AP	0.285	0.60	7.376	0.01
ML	0.806	0.38	0.008	0.93
Area	1.165	0.29	1.841	0.19
(2) EC floor				
AP	0.767	0.39	4.744	0.04
ML	0.090	0.77	1.502	0.24
Area	0.142	0.71	0.009	0.92
(3) EO foam				
AP	0.016	0.90	3.209	0.09
ML	0.979	0.34	1.198	0.29
Area	0.312	0.58	2.125	0.16
(4) EC foam				
AP	0.159	0.69	1.756	0.20
ML	0.968	0.34	0.615	0.44
Area	0.250	0.62	1.232	0.28

**Table 3:** Comparison of postural sway between pretest and posttest in control groups by using one-tailed, paired *t*-test at *α* = 0.05.

	Paired differences					*t*	df	*p* (1-tailed)
	Mean	Std. deviation	Std. error mean	95% confidence interval of the difference				
				Lower	Upper			
Pre-/posttest AP, EO floor	-10.90	36.12	11.42	-36.74	14.94	-0.954	9	0.18
Pre-/posttest ML, EO floor	-1.70	23.42	7.40	-18.45	15.05	-0.230	9	0.41
Pre-/posttest area, EO floor	-547.20	2047.48	647.47	-2011.87	917.47	-0.845	9	0.21
Pre-/posttest AP, EC floor	-11.80	42.08	13.30	-41.90	18.30	-0.887	9	0.20
Pre-/posttest ML, EC floor	18.80	37.29	11.79	-7.87	45.47	1.594	9	0.07
Pre-/posttest area, EC floor	1027.60	3059.38	967.46	-1160.95	3216.15	1.062	9	0.16
Pre-/posttest AP, EO foam	-17.40	38.51	12.18	-44.95	10.15	-1.429	9	0.09
Pre-/posttest ML, EO foam	5.60	17.43	5.51	-6.86	18.06	1.016	9	0.17
Pre-/posttest area, EO foam	-337.20	2977.63	941.61	-2467.27	1792.87	-0.358	9	0.36
Pre-/posttest AP, EC foam	3.90	45.14	14.27	-28.39	36.19	0.273	9	0.39
Pre-/posttest ML, EC foam	-14.90	36.47	11.53	-40.98	11.18	-1.292	9	0.11
Pre-/posttest area, EC foam	-505.20	4625.22	1462.62	-3813.88	2803.48	-0.345	9	0.37

**Table 4:** Comparison of postural sway between pretest and posttest in experimental groups by using one-tailed, paired *t*-test at *α* = 0.05.

	Paired differences					*t*	df	*p* (1-tailed)
	Mean	Std. deviation	Std. error mean	95% confidence interval of the difference				
				Lower	Upper			
Pre-/posttest AP, EO floor	22.40	22.01	6.96	6.66	38.14	3.219	9	0.01
Pre-/posttest ML, EO floor	7.50	21.70	6.86	-8.03	23.03	1.093	9	0.15
Pre-/posttest area, EO floor	1164.10	1905.82	602.67	-199.24	2527.44	1.932	9	0.04
Pre-/posttest AP, EC floor	6.90	29.26	9.25	-14.04	27.83	0.746	9	0.24
Pre-/posttest ML, EC floor	10.90	17.98	5.69	-1.97	23.77	1.917	9	0.04
Pre-/posttest area, EC floor	574.10	2022.86	639.69	-872.97	2021.17	0.897	9	0.39
Pre-/posttest AP, EO foam	14.00	42.92	13.57	-16.70	44.70	1.032	9	0.16
Pre-/posttest ML, EO foam	20.90	28.15	8.90	0.76	41.04	2.348	9	0.02
Pre-/posttest area, EO foam	1816.40	2729.03	863.00	-135.83	3768.63	2.105	9	0.03
Pre-/posttest AP, EC foam	16.10	27.56	8.72	-3.62	35.82	1.847	9	0.05
Pre-/posttest ML, EC foam	9.50	29.45	9.31	-11.57	30.57	1.020	9	0.17
Pre-/posttest area, EC foam	1871.70	4393.56	1389.37	-1271.26	5014.66	1.347	9	0.11

## Data Availability

The data used to support the findings of this study are included within the article.
